# Advancing equity in global health: a call for collaborative research partnerships

**DOI:** 10.3389/frhs.2025.1541472

**Published:** 2025-05-21

**Authors:** Mirlene Perry, Kim Madundo, Shanti Narayanasamy, Brandon Knettel

**Affiliations:** ^1^ Duke University School of Nursing, Durham, NC, United States; ^2^ Duke Global Health Institute, Durham, NC, United States; ^3^ Department of Psychiatry and Mental Health, Kilimanjaro Christian Medical Centre, Moshi, Tanzania; ^4^ Department of Psychiatry, KCMC University, Moshi, Tanzania; ^5^ Department of Medicine, University of Melbourne Austin Hospital, Melbourne, VIC, Australia; ^6^ Duke Center for Global Mental Health, Durham, NC, United States

**Keywords:** global health, equity, partnerships, decolonization, equitable partnerships

## Abstract

Addressing global health challenges require collective efforts and equitable partnerships that share knowledge and resources across borders. It also requires equitable partnerships among local researchers and research participants to prevent reproducing decolonization within country. This perspective explores the multifaceted nature of partnerships in global health, examine the benefits of equitable partnerships, highlights challenges like power imbalances but also newer efforts to decolonize global health research. It also advocates for a more ethical approach in global health research to address structural inequities and promote long-term sustainability in global health initiatives across the global South.

## Introduction

Addressing complex and pressing challenges in global health demands collective efforts (1). The principle of equitable partnerships in global health research has gained significant recognition (2, 3) because equitable partnerships enable “exchange of learning and resources” (4) across borders. In this perspective, we describe the multifaceted nature of partnerships in global health, examine the benefits of equitable partnerships, acknowledge challenges, and advocate for a more ethical and equitable approach.

## Background

This research team consists of a Haitian-born doctoral student who has lived in the United States and worked as a nursing professional for the past 15 years; a biracial (Black-Western European) psychiatrist and researcher who has lived in both the global North (England) and South, and now lives and works in the South (Tanzania); a brown, queer, clinician-researcher of South Asian heritage, born in Australia living on Wurundjeri land where sovereignty was never ceded, who has been championing decolonization for several years and teaches graduate-level courses; and a U.S.-born licensed psychologist who has been working in East Africa for 16 years and teaches graduate-level courses in the United States that consider diverse topics of global mental health, including decolonization. We acknowledge our privilege to pull our experiences together to work on this project.

For this perspective, decolonization is defined as “an ongoing social process by which formerly colonized societies assert political independence, dismantle colonial structures, and reclaim cultural, economic, and social autonomy by challenging colonial ideologies, restoring indigenous knowledge systems, and restructuring institutions to reflect local values and identities” (5). In this context, a global health researcher is rather a guest, not an authority. Equity is defined as “a propositional value judgement about how fair or how just social systems, structures, institutions, processes, or policies might be” (6) and power as “capacity of actors to mobilise means to achieve ends” (7).

At their outset, international health programs were embedded within colonialism in which unequal partnerships existed between white European settlers and the people in the land they occupied (2, 3) but also within non-consensual medical interventions and vaccine experiments on colonized populations. For example, the latter involved managing sleeping sickness, mass vaccinations campaigns with yellow fever in French West Africa (8), and other parts of the world including Haiti (9) leading to mistrust. This structural inequality was rooted in injustices in which the colonizer dominated the colonized, extracted resources, and controlled knowledge production leading to a perpetual cycle of disparities (10). Here, we refer to settlers, colonizers, and those directed programs from the global North as the global North and the Indigenous peoples (colonized) as the global South. The institutions of colonialism helped the global North to become more affluent than the global South, extending beyond socio-economic status to ensure better health outcomes in the North (11, 12).

In recent years, inequities in affluence and health have persisted, and in some cases worsened, despite economic development in both regions, technological advances, and marked increases in global interconnectedness (11, 12). Global North organizations and researchers, while contributing resources for the development of the global South, often dictated the agenda without adequate input from the global South. This dynamic, primarily perceived as a charitable cause in the global North (2), underscored power imbalances, perpetuated a sense of white saviorism (13), and raised the expectation of a more submissive role from the global South (14). A stance of charity by the global North needs to be recentered towards global justice and empowerment of global South researchers, institutions, and communities to become equal partners (13) with a focus on shared intent and dignity.

Beyond addressing power dynamics between global North and South, decolonization also requires global South institutions to assert greater agency over their own affairs (14, 15). This includes engaging in critical introspection to avoid reproducing colonial legacies through local hierarchies, and valuing the voices and roles of all participants in global health research. One pathway forward is through fostering meaningful community engagement and participatory approaches whenever feasible (16). These practices help prevent the replication of global North/South power imbalances within global South contexts, especially between researchers and marginalized community members who often serve as passive participants. Research participants should be regarded not merely as subjects, but as equal partners and co-leaders in the research process. This paradigm shift supports deeper community involvement and encourages relational, rather than transactional, forms of engagement (17). Hence, a more inclusive and more collaborative model, one that involves researchers from all partner sites, is needed to address power asymmetries and promote sustainability in the global South.

## Power imbalances in academia

In the academic realm, power imbalances persist, and they are fueled by a mismatch of resources vs. needs in the global South (13). Imbalances stemming from resource disparities hinder the equitable and just distribution of benefits in both regions, further maintaining a cycle of dependence and white saviorism. Global North partners may be incentivized by funding acquisition and the accolades that come from program development, rather than the benefits that come from long-term, impactful, and sustainable development. This may create conditions where global North partners may be less committed to provide the long-term effort and resources required for empowerment of their global South partners. Humility, sustainability, and long-term mutual benefit should be at the core of these partnerships in order to promote equity and fairness (2).

Generating and sharing knowledge through global health research is crucial for advancing global health. However, equitable collaboration must be emphasized in every facet of such endeavors. Efforts co-led by researchers from both regions have enhanced detection of diseases and their treatments, such as HIV, Ebola, COVID-19, and other health issues (18–23). The pooling of expertise enhances the effectiveness of interventions and sustainable health outcomes. However, global South researchers may not always receive due respect and acknowledgement for their contributions, as exemplified by the case of Dr. Jean-Jacques Muyembe, a Congolese physician instrumental in identifying Ebola (18).

With his keen observation and dedication to improving patient outcomes in his native land, Dr. Muyembe set out to identify the cause of a new symptom in his patients, hemorrhagic fever in 1976 (18). Lack of resources prevented him from conducting his research locally. In his quest to identify the nature of this new symptom, he decided to collaborate with global North researchers towards better understanding this new phenomenon. Upon identifying the new virus, Ebola, global North researchers took the credit for the work (18). This situation happened because Dr. Muyembe is a member of the global South and lacked the resources to answer his research question.

### Academic authorship

This lack of visibility extends to researchers in the academic realm. The structure of the academic system in the global North poses challenges for global health researchers, both new and seasoned, to relinquish their positions in academic papers (24, 25). Academic promotions often place considerable weight on the number of published articles and the authors' positions within them (26). This framework may make it challenging for global North researchers to co-lead or take a supporting role in manuscript writing with global South counterparts.

Authorship debates can also be compounded by the fact that financial resources often originate from the global North, limiting the voice of global South researchers. Moreover, academic journals often set or adopt structured standards for authorship (27). Although these standards are typically established to prevent “gift authorship” or other forms of including authors who have not made a substantial contribution to the work, the standards may also fail to encourage acknowledgement of global South contributions to research that does not fall within the conventional understanding of authorship in the global North. For example, invaluable efforts in community outreach and coalition building may not be recognized as research contributions worthy of authorship.

In academic medical writing for English language journals, global South researchers may be at a disadvantage when English is not their first language. Global North institutions could help to mitigate this problem by establishing a mentor-mentee system between researchers from the global North and South, enabling the latter group to take on roles beyond data collection, and promoting capacity building and equity in academic authorship (28). Global North institutions and researchers should plan and budget for additional time and training resources to partner with global South authors on manuscript writing, professional development, leadership training, and other forms of capacity building.

The ability to convey research findings is critical, aligning with Abimbola's concept of using a local gaze instead of a foreigner's gaze (29). A foreigner's gaze restricts researchers in the global South from presenting their findings authentically, which also hinders effective implementation (30, 31). Global health researchers from the North should employ reflexivity, examining their motives and assumptions, to enhance equity in their work (29). Achieving equitable inclusion of researchers from both regions is vital for better representation of local perspectives in the conduct of research and resulting publications (29). Naidoo's report on the underrepresentation of African authors during the COVID-19 pandemic is a prime example of the failure of the academic enterprise to support global South researchers on important research topics for which they have special expertise (32).

## Ethics and cultural humility

Cultural differences, along with different ethical standards across different global settings, pose additional obstacles to equitable partnerships (33, 34). Academic ethics must take precedence in global health research collaborations, ensuring that partnerships are founded on principles of respect, transparency, and cultural humility. Some academic journals encourage the inclusion of global South researchers with lived experiences to submit research manuscripts, and encourage global North authors to include global South partners as co-authors (35) but many global health journals do not have such policies. Others call for moving global South journals in the center (36), promoting open access to enhance inclusion and diversity (37), reducing publication fees for global South researchers could also contribute to this dialogue. More journals need to follow suit. Prioritizing the autonomy and well-being of local academic communities is paramount, empowering local researchers to use their own gaze to present their findings (29). In the global North, cultivating cultural humility and a decolonizing perspective is critical for the success of collaborative efforts. Understanding the nuances of local contexts, beliefs, and practices is required for effective global health research partnerships. Fostering cultural humility and building trust within academic communities through genuine partnerships will contribute to empowerment in the global South.

## Elite capture

Elite capture of decolonization was defined by Krugman as “coopting and reconfiguration of radical liberatory theories and concepts then used by elites for their own gain” (3). Elite capture is another aspect in need of reform for advancing partnerships in global health research. Institutions in the global North have signed on to efforts focused on diversity and decolonization without taking into account the brutal history of colonialism (3). Academic institutions giving lip service to efforts to delink this historical trauma from decolonization, while in reality doing the bare minimum to acknowledge and address past and current transgressions, is an example of elite capture. Resisting elite capture calls for acknowledging ongoing transgressions, taking bold steps towards reparations through funding mechanisms, and moving away from the perception of the global North as “white saviors” (29).

Funders and other global health decision makers should consider prioritizing equity, inclusivity, and shared-decision-making to improve partnerships in global health research (3) by gradually and purposively providing research funds to global South institutions that involve sub-contracting with global North researchers instead of the other way around. Grant funding should emphasize capacity-building initiatives and promote active involvement and recognition of researchers from the global South in the literature. These steps can help to rectify power imbalances, foster more meaningful collaborations, and build a more equitable, effective, and sustainable landscape for global health research.

## Double agency in global health research

In the context of decolonization, double agents are individuals originating from the global South who are now employed by or otherwise representing institutions in the global North (38). The role of these individuals in global health research is complex. Their dual identities, experiences, and perspectives can serve as bridges between both regions, and can lend support to their efforts to use global North resources to rectify the imbalances produced by colonialism. At the same time, double agents must be thoughtful in navigating their representation of the global North by not adopting or perpetuating colonial approaches to global health research. Double agents may also have contributed to the “brain drain”, where talented individuals leave the global South for professional and personal advancements in the global North. Nonetheless, leveraging double agents as brokers and mediators can facilitate meaningful conversations and progress toward equity as a result of their unique positionality (29).

We also want to recognize some newer efforts happening in the global North and the Global South. Certain global North funders are requiring applicants to discuss equity in research partnerships as part of grant applications (39, 40). In Africa for example, some African-based funders are actively working to rectify power imbalances through self-agency and epistemic justice pulling research funds at national or regional levels for expanding indigenous non-governmental organizations, shifting power to local actors, promoting indigenous knowledge systems, and supporting self-determined development. For example, the African Alliance's Decolonise R&D Fellowship challenges dominant global research norms by centering African voices in health and development (41). On the philanthropic side, Liberation Alliance Africa advocates for decolonial feminist funding practices (42), MoFund Africa enhances transparency and accessibility for African NGOs (43), while EPIC-Africa promotes critical consciousness to transform donor-grantee relationships (44). More similar actions should follow suite.

## Conclusion

Equitable partnerships between the global North and South are essential for advancing the field of global health. A commitment to reflexivity, ethical conduct, and cultural humility from global North researchers and institutions can help harness the full potential of these partnerships and at the same time recognize and address power imbalances. Now, more than ever before, is the time to regroup, work together, and invite each other at the table to advance global health equity whether one is in the global South or North. Within-country dynamics are equally important. One question to ask ourselves as we go through global health research is “how would I feel if I was on the receiving end of a research partnership”? Or “what can I do to promote genuine and collaborative partnerships in global health research”? A genuine and collaborative approach between researchers from the global North and South as well as local research participants within country could lead us all to improved research outcomes as each partner will appreciate being heard, but most importantly being part of the solution while we work incessantly to attain or contribute to the overarching goal of achieving global health equity.

## Data Availability

The original contributions presented in the study are included in the article/Supplementary Material, further inquiries can be directed to the corresponding author.
